# Single radial haemolysis compared to haemagglutinin inhibition and microneutralization as a correlate of protection against influenza A H3N2 in children and adolescents

**DOI:** 10.1111/irv.12450

**Published:** 2017-03-16

**Authors:** Biao Wang, Margaret L. Russell, Angela Brewer, Jennifer Newton, Pardeep Singh, Brian J. Ward, Mark Loeb

**Affiliations:** ^1^ Department of Pathology and Molecular MedicineMcMaster UniversityHamiltonONCanada; ^2^ Department of Community Health SciencesCumming School of MedicineUniversity of CalgaryCalgaryABCanada; ^3^ Research Institute of the McGill University Health CenterMontrealQCCanada; ^4^ Department of MedicineMcMaster UniversityHamiltonONCanada; ^5^ Department of Clinical Epidemiology and BiostatisticsMcMaster UniversityHamiltonONCanada; ^6^ Michael G. De‐ Groote Institute for Infectious Disease ResearchMcMaster UniversityHamiltonONCanada

**Keywords:** children and adolescents, correlate of protection, haemagglutinin inhibition, influenza A H3N2, microneutralization, single radial haemolysis

## Abstract

**Background:**

Serum antibodies are often used as correlates of protection for influenza. Three commonly used serological assays for detecting influenza‐specific serum antibodies are single radial haemolysis (SRH), haemagglutinin inhibition (HAI) and microneutralization (MN). However, here are limited data on SRH as well as HAI and MN as correlates of protection against influenza in children and adolescents. There are also limited data that compare SRH to HAI and MN.

**Objectives:**

We sought primarily to understand how SRH titres correlate to protection against influenza infection in children and adolescents. We also compare SRH to HAI and MN.

**Methods:**

Of 732 healthy Hutterite children and adolescents aged between 3 and 15 years were enrolled from Saskatchewan and Alberta, Canada, in the 2008‐2009 flu season. Blood samples were drawn from participants at baseline and between 3 and 5 weeks post‐vaccination. Serum antibodies against seasonal H3N2 influenza were measured by SRH, HAI and MN assays.

**Results:**

The estimates of protective efficacy fluctuated when the cut‐off SRH values increased. The correlation between HAI and SRH titres was 0.53 (*P*<.01); between MN and SRH 0.82 (*P*<.01); and between HAI and MN 0.50 (*P*<.01). Sixteen per cent of participants had SRH titres below the detection limit, compared to 7% and 34% for the MN and HAI assays.

**Conclusions:**

SRH had the worst correlation with protection against seasonal H3N2 in children and adolescents compared to MN and HAI. SRH, HAI and MN titres were significantly correlated with each other. SRH was less sensitive than MN but more sensitive than HAI.

## 1 Introduction

Serum antibody titres are often used as correlates of protection for influenza.^1^ Three commonly used serological assays for detecting influenza‐specific serum antibodies are single radial haemolysis (SRH), haemagglutinin inhibition (HAI) and microneutralization (MN), of which SRH and HAI are the most widely used. The SRH assay utilizes antibody diffusion in agar gel to measure the antibody content of test sera.^2^, ^3^ This method quantifies antibodies by measuring the areas of haemolysis, which, mediated by complement and induced by the antibody‐antigen complex, are proportionated to the concentration of antibodies. The HAI assay detects antibodies that can prevent agglutination of erythrocytes.^4^ The antibody concentration is quantified as the reciprocal of the highest serum dilution (titre) that inhibits agglutination using a standard assay. MN technique is a form of viral neutralization (VN) that commonly uses cultured Madin‐Darby canine kidney (MDCK) cells.^5^ The method detects antibodies capable of neutralizing the ability of the virus to enter or replicate in mammalian cells. The quantity of the antibodies is expressed as the reciprocal of the highest serum dilution that induces at least 50% of cytopathic effect in mammalian cell cultures.

By convention, a SRH titre ≥25 mm^2^ and an HAI titre of ≥1:40 are considered to be associated with a 50% or higher protection against influenza, while in contrast there are no established thresholds for the MN assay.^6^ The cut‐off values for SRH and HAI titres were previously adopted by the Committee for Proprietary Medicinal Products for Human Use (CHMP) as criteria that should be considered for influenza vaccine licensure in Europe.^7^ These thresholds however were derived from limited challenge studies in healthy adults performed decades ago and the transferability of these thresholds to other subpopulations, such as children, has never been established. In recent years, there has been growing recognition of the limitation of these established criteria which has led to the abolition of these criteria in the newly developed guideline for influenza vaccine licensing effective February 2017 by CHMP.^8^, ^9^ Until now, there has still been a relative lack of data on all three aforementioned serologic assays as correlates of protection against influenza in young age groups.

Few studies have compared all three assays together on a common serum base. In fact, we found only two such studies and they were limited to equine influenza viruses. In the first study, SRH, HAI and MN tests were performed on sera from horses immunized against two equine influenza viruses including Prague (H7N7) and Miami (H3N8) strains.^10^ They found that MN titres of individual sera were highly correlated with the HAI titres (0.96 for Prague strain and 0.91 for Miami strain), while correlations between SRH and HAI were much lower (0.75 for Prague strain and 0.77 for Miami strain). The correlations between SRH and MN were similar (0.77 for Prague strain and 0.75 for Miami strain). The second study reported correlations between the three assays ranged from 0.83 to 0.96 based on sera from 41 horses during an epidemic associated with four equine influenza viruses including Saskatoon (H3N8), Kentucky (H3N8), Miami (H3N8) and Prague (H7N7) strains.^11^ Although there have been studies that compare two of the three assays separately based on human influenza viruses,^12^, ^13^, ^14^ to the best of our knowledge there has not been a study comparing all three assays together on a common serum base.

We previously reported that for influenza A H3N2, MN is superior to HAI in terms of showing correlates of protection in children and adolescents.^15^ In this paper, we sought primarily to understand how SRH titres correlate to protection against influenza A H3N2 infection in children and adolescents. We also compared SRH to HAI and MN.

## 2 Materials and Methods

### 2.1 Participants and serum samples

In this study, we enrolled healthy Hutterite children and adolescents aged 3‐15 years from Hutterite colonies in Saskatchewan and Alberta in the 2008‐2009 influenza season who participated in a cluster randomized trial (clinicaltrials.gov: NCT00877396; isrctn.org: ISRCTN15363571).^16^ In this trial, participants were randomly assigned by colonies to two study groups—trivalent influenza vaccine (TIV) group and the hepatitis A vaccine (HAV) group. There were 1773 participants from 22 colonies assigned to TIV group and 1500 participants from 24 colonies assigned to HAV group in the 2008‐2009 influenza season. Healthy children and adolescents aged 3‐15 years from these two study groups received either TIV or the HAV depending on which study group their Hutterite colony was randomized to. 502 children and adolescent participants in TIV group received TIV and 445 children and adolescent participants in HAV group received HAV in the trial. For this study, we enrolled and obtained specimens from 732 children and adolescents participants from the RCT trial. Of these, 348 were from TIV group and 384 were from HAV group. The three antigens included in the TIV were A/Brisbane/59/2007 (H1N1)‐like, A/Brisbane/10/2007 (H3N2)‐like and B/Florida/4/2006‐like viruses, all of which were well matched with circulating strains in Canada during the 2008‐2009 flu season. Blood samples were drawn from participants at baseline and between 3 and 5 weeks post‐vaccination. Active surveillance was conducted twice weekly during the influenza season (28 December 2008 to 23 June 2009) and those with two or more signs or symptoms compatible with influenza were tested by RT‐PCR of nasal swabs.^16^

### 2.2 Antibody tests

SRH assays were prepared as described in Trombetta et al.^1^ In brief, the A/Brisbane/10/2007 (H3N2)‐like virus was obtained from the British Columbia Centre for Disease Control (Vancouver, BC) and virus stocks were grown in Madin‐Darby canine kidney (MDCK) cells in the FBS‐free media MegaVir. A fresh turkey erythrocyte suspension (Lampire) was mixed with diluted virus antigen at a concentration of 2000 haemagglutinating units (HAU)/mL and then incubated at 4°C for 20 minutes. Then, 2.5 mmol/L CrCl_3_ was added to the mixture and incubated for 10 minutes at room temperature. The suspension was then centrifuged at 550 × *g* for 15 minutes. The supernatant was removed and the pellet was resuspended with phosphate‐buffered saline. Prior to the incubation of the antigen‐erythrocyte mixture, a solution of 1.5% agarose was prepared and kept in a water bath at 47.5°C. The resuspended erythrocyte‐antigen mixture followed by guinea pig complement (Sigma) was added to the agarose gel. The mixture was then spread onto plates (7.2 cm × 2.3 cm) and incubated for 30 minutes at room temperature and then for 30 minutes at 4°C. Twenty holes were then made with a 2.25‐mm calibrated punch into each plate. Serum samples and controls (6 μL each) were seeded into the holes and returned to 4°C immediately. The plates were then incubated for 18 hours at 4°C in a humid chamber. The plates were then incubated for 90 minutes in the humid chamber at 37°C and the diameter of each haemolysis halo was read in millimetres using the TG Calibrating Viewer.

The HAI assay was performed as previously described using turkey erythrocytes and reference antigens for A/Brisbane/10/2007 (H3N2)‐like viruses.^17^

MN titres were determined as previously described.^15^ Briefly, we purchased the A/Brisbane/10/2007 (H3N2)‐like virus from BEI (Manassas, VA) and prepared influenza virus stocks in MDCK cells. Sera were heat‐inactivated (56°C for 30 minutes) and stored at −20°C until use. Twofold serial dilutions of serum starting at 1:10 in MegaVir were incubated with 100 50% tissue culture infective doses (TCID50) of virus for 2 hours at 37°C with 5% CO_2_. We then transferred the serum and virus to plates containing a monolayer of MDCK cells in MegaVir medium with 1 × TPCK (tolylsulfonyl phenylalanyl chloromethyl ketone)‐treated trypsin. After 3 hours at 37°C with 5% CO_2_, we refreshed the medium in each well in the plates with MegaVir medium with 0.75 × TPCK‐treated trypsin. After 4‐5 days of incubation, we observed cells for the presence of cytopathic effect. We defined the MN titre value as the highest dilution to retain a confluent cell monolayer. We assigned a value of 1:5 for MN titres below the limit of detection (<1:10) for statistical analysis.

For all three assays, we used internally sourced and validated positive and negative controls. We also included back titration of virus for HAI and MN assays.

### 2.3 Statistical analysis

We evaluated the correlation of protection against influenza of SRH titre by calculating protective effectiveness at each titre threshold using Cox's proportional hazards model,^16^ adjusting for clustering using a robust sandwich estimator. The protective effectiveness was defined as (1‐hazard ratio) × 100%. We did the same calculation for HAI titre and MN titre thresholds. Traditionally, a HAI titre of 1:40 is accepted in literature as 50% protective against influenza infection.^7^ We used 50% protective efficacy as our reference point. We calculated Spearman rank correlation coefficients for antibody levels comparing SRH and HAI antibody levels to MN antibody, levels after vaccination, correcting for ties in ranking.^18^, ^19^ We also used linear regression to estimate the associations between SRH titre value and log‐transformed HAI and MN titre values. All analyses were performed in R version 3.0.1 (R Foundation, Vienna, Austria).

## 3 Results

We detected seven (2.0%) PCR‐confirmed seasonal H3N2 cases in participants who had received TIV influenza vaccine and 19 (4.9%) in participants who had received hepatitis A vaccine.

The distribution of pre‐ and post‐vaccination antibody responses tested by SRH, HAI and MN assays is shown in Table 1. As expected, there was difference between pre‐ and post‐titre values in influenza group measured by the three assays, whereas the pre‐ and post‐titre values were similar.

**Table 1:** Distribution of Pre‐ and Post‐vaccination Titre Values of Single Radial Haemolysis, Haemagglutinin Inhibition and Microneutralization Assay

		Influenza Group N=384		Hepatitis A Group N=348	
		Pre Mean ± SD	Post Mean ± SD	Pre Mean ± SD	Post Mean ± SD
SRH a	All age	23.28±17.89	56.84±22.24	20.71±17.31	22.77±18.61
	3‐6 years	26.76±19.39	53.95±21.84	26.65±18.73	26.50±20.01
	7‐9 years	28.00±17.84	64.69±18.34	21.35±17.01	24.53±21.87
	10‐15 years	18.85±16.07	53.84±23.57	17.41±15.96	20.19±15.51
HAI b	All age	3.70±3.13	5.54±3.32	3.10±2.72	2.54±2.63
	3‐6 years	4.41±3.25	6.06±3.05	3.72±3.06	2.46±2.76
	7‐9 years	4.85±2.91	6.07±3.33	2.66±2.79	2.63±2.85
	10‐15 years	2.61±2.82	4.94±3.39	3.03±2.47	2.53±2.45
MN b	All age	3.60±2.53	7.15±2.40	3.42±2.51	3.81±2.60
	3‐6 years	4.11±2.99	6.70±2.75	4.20±2.93	4.29±3.07
	7‐9 years	4.41±2.25	7.98±1.70	3.09±2.48	3.62±2.73
	10‐15 years	2.89±2.22	6.92±2.42	3.21±2.24	3.71±2.27

SD, standard deviation; SRH, single radial haemolysis; HAI, haemagglutinin inhibition; MN, microneutralization.

a The unit for SRH titre values is mm^2^.

b HAI and MN titre values are log‐transformed through the formula: [log_2_(titres/10) +1].

**Figure 1:**
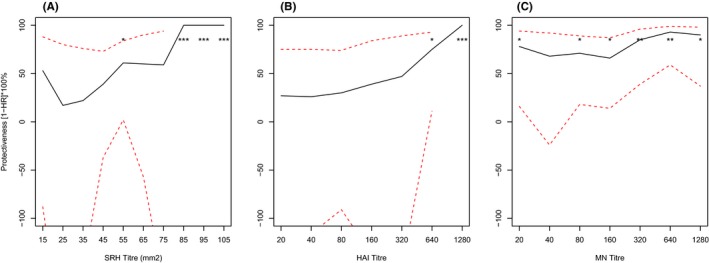
**SRH** **titre value has the worst correlation with protection against influenza, as the estimates of protective efficacy fluctuated when the cut‐offs of** **SRH** **titre value increased. The estimates of protective efficacy for** **MN** **were relatively stable and consistently higher than those of both** **SRH** **and** **HAI** **.** Protective effectiveness against PCR‐confirmed influenza was compared at different SRH, HAI and MN titre cut‐offs for seasonal H3N2 (A/Brisbane/10/2007). The hazard ratio (HR) represents the risk at cut‐offs greater than or equal to a given titre, relative to levels less than the cut‐off, and was calculated using Cox's proportional hazards model, adjusting for participant colony using a robust sandwich estimator. Dotted lines represent the 95% confidence interval and *P*‐values were calculated using standard error estimates from the regression model. **, *P*<.01; *, *P*<.05.

We found that, for SRH titres [Figure 1A], the estimates of protective efficacy fluctuated when the cut‐off SRH values increased. The estimates first dropped before the SRH titre value of 25 mm^2^. The estimates then increased with the increase in SRH cut‐off values and reached a plateau of 60% at a cut‐off value of 55 mm^2^. The estimates increased again after a cut‐off value of 75 mm^2^ and reached a maximum at 85 mm^2^. In contrast, for HAI titre (Figure 1B), estimates of protective effectiveness rose continuously with an increase in titre value and an HAI titre value of >1:640 correlated to a protective effectiveness >50%. The estimates of protective effectiveness of MN titres [Figure 1C] were always >50% and consistently higher than those of HAI titres.

**Figure 2:**
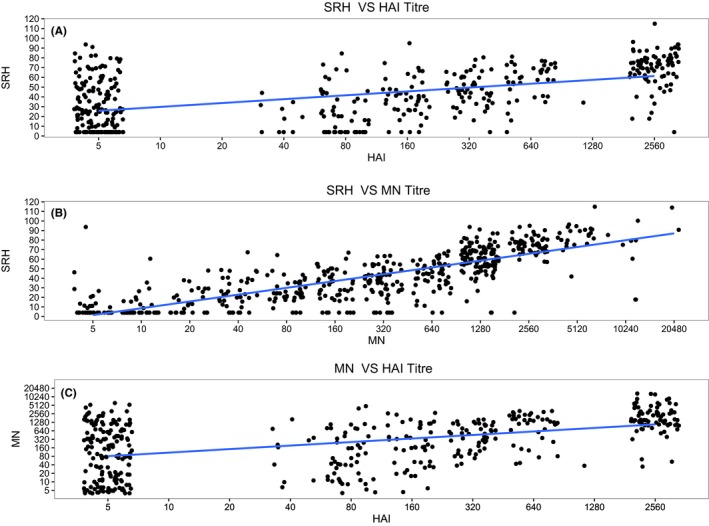
**Relationship between haemagglutinin inhibition (** **HAI** **) titres and single radial haemolysis (** **SRH** **) titres (A), between microneutralization (** **MN** **) titres and** **SRH** **(B) and between** **HAI** **titre and** **MN** **titre for seasonal influenza H3N2.**

Median titres (25th–75th quartiles) for these three methods were as follows: SRH, 42 (19–64) mm^2^; HAI, 80 (5–640); MN, 320 (40–1280). Sixteen per cent of participants had SRH titres below the limit of detection (<3.99 mm^2^); 34% had HAI titres below the limit of detection (<1:10); and 7% had MN titres below the limit of detection (<1:10). The Spearman rank correlation between HAI and SRH titres was 0.53 (*P*<.01); between MN and SRH 0.82 (*P*<.01); and between HAI and MN titres 0.50 (*P*<.01). Based on linear regression, an HAI titre of 1:40 corresponds to a MN titre of approximately 1:160, and to a SRH titre of 38 mm^2^, respectively (Figure 2).

## 4 Discussion

The major finding of this study is that the threshold values for SRH titres as well as HAI titres corresponding to 50% protection against influenza in children ≥3 years and adolescents ≤15 years are much higher than the criteria recommended by CHMP (SRH, >55 mm^2^ vs >25 mm^2^; HAI, >1:640 vs >1:40).^3^ This result supports the need for the development of age‐specific criteria for these assays.

One recent study also challenged the applicability of these criteria in Children.^20^ The authors reported that for children ≤6 years of age, a cut‐off of 1:110 HAI titre value many be used to predict the conventional 50% clinical protection rate for seasonal H3N2. Our result of 1:640 may be an overestimate as the sample size in our study was limited and the result is not statistically significant. Currently, there are no established correlates of protection for young age groups in all the three assays. Nevertheless, the new CHMP guideline has abolished the traditional criteria with the recognition that relying on these criteria of measuring protective effectiveness might not be appropriate for various situations and this opens the opportunities to advance the field on correlates of protection in various subpopulations against different influenza strains.^9^

Another key finding was that SRH appears to have the worst correlation with protection against influenza H3N2, as the estimates of protective efficacy fluctuated when the cut‐off SRH values increased. The estimates of protective efficacy for MN were relatively stable and consistently higher than those of both SRH and HAI. Our results show that among the three assays, MN titres offer the best correlate of protection against influenza H3N2. The likely explanation, as suggested by Trombetta et al.,^6^ is that the MN assay detects functional antibodies able to neutralize virus (vs HA binding in the HAI assay and complement fixation in the SRH assay), and therefore measures a greater proportion of the antibodies implicated in protection.

We found the SRH assay to be less sensitive than the MN assay but more sensitive than HAI, as 16% of participants had SRH titres below the detection limit, compared to 7% and 34% for the MN and HAI assays, respectively. Previous studies have shown that MN assay in more sensitive than HAI assay.^1^, ^3^, ^6^, ^21^ However, we could not find any data that compared SRH and MN in terms of sensitivity. The reported relationship in literature between HAI and SRH appears variable. Reports by Mancini et al.^11^, ^12^ suggest that SRH may be more sensitive for detecting antibodies against influenza B than influenza A viruses, while the study by Yamagishi et al.^10^ suggests that the sensitivity of SRH test is lower than that of HAI, at least for equine influenza virus.

We also found that SRH, HAI and MN titres were all significantly correlated, and the correlation coefficient between SRH and MN (0.82, *P*<.01) was much larger than that between SRH and HAI (0.53, *P*<.01) and between HAI and MN (0.52, *P*<.01). Morley et al.^11^ have reported correlations between these three assays for pre‐exposure and changes in antibody titres ranging from 0.83 to 0.96 for equine influenza virus (eg H3N8). Our results for the correlation between SRH and MN are similar but correlations between MN and HAI and between HAI and SRH are much lower. This discrepancy warrants further investigation.

The strength of our study is that it provides much needed information regarding SRH as a correlate of protection against influenza. The comparison among the three methods also contributes to the literature considering the limited data available. One limitation of this study is that we had a relatively small sample size considering that sufficient post‐vaccination serum for all three assays (ie HAI, SRH and MN) was available for only 15 of the RT‐PCR‐confirmed cases. Another limitation is that our surveillance did not detect natural exposures (symptomatic or asymptomatic).
